# Large anomalous Hall effect in the chiral-lattice antiferromagnet CoNb_3_S_6_

**DOI:** 10.1038/s41467-018-05756-7

**Published:** 2018-08-16

**Authors:** Nirmal J. Ghimire, A. S. Botana, J. S. Jiang, Junjie Zhang, Y.-S. Chen, J. F. Mitchell

**Affiliations:** 1 Materials Science Division, Argonne National Laboratory, 9700 South Cass Avenue, Argonne, IL 60439 USA; 2 ChemMatCARS, The University of Chicago, Argonne, IL 60439 USA

## Abstract

An ordinary Hall effect in a conductor arises due to the Lorentz force acting on the charge carriers. In ferromagnets, an additional contribution to the Hall effect, the anomalous Hall effect (AHE), appears proportional to the magnetization. While the AHE is not seen in a collinear antiferromagnet, with zero net magnetization, recently it has been shown that an intrinsic AHE can be non-zero in non-collinear antiferromagnets as well as in topological materials hosting Weyl nodes near the Fermi energy. Here we report a large anomalous Hall effect with Hall conductivity of 27 Ω^−1^ cm^−1^ in a chiral-lattice antiferromagnet, CoNb_3_S_6_ consisting of a small intrinsic ferromagnetic component (≈0.0013 *μ*_B_ per Co) along *c*-axis. This small moment alone cannot explain the observed size of the AHE. We attribute the AHE to either formation of a complex magnetic texture or the combined effect of the small intrinsic moment on the electronic band structure.

## Abstract (web summary)

Anomalous Hall effect (AHE) in antiferromagnets is intriguing and requires further understanding. Here the authors report large AHE in a chiral-lattice antiferromagnet CoNb_3_S_6_ of which the origin can be due to complex magnetic texture or broken time-reversal symmetry on the electronic band structure.

## Introduction

The anomalous Hall effect (AHE) is a signature of emergent electromagnetic fields in solids that affect the motion of the electrons, and hence it has been a recent topic of intense study in the context of the topological materials^1,2^. The Hall effect in general is an intrinsic property of a conductor due to the Lorentz force experienced by the charge carriers. In systems with spontaneously broken time-reversal symmetry, an additional contribution, independent of the Lorentz force, is observed, the AHE^1^. AHE was first observed in ferromagnets where its origin lies in the interplay between spin–orbit coupling (SOC) and magnetization. Reformulation of the SOC-induced intrinsic mechanism of AHE in ferromagnets to the Berry phase curvature in momentum space has been fruitful in predicting and describing the AHE in several other systems, including Weyl (semi)metals^3^, non-collinear antiferromagnets^4^, non-coplanar magnets^5–7^, and other nontrivial spin textures^8–11^. Recent observations of the large anomalous Hall effect in metals with possible Weyl^12–14^ and massive Dirac fermions^15,16^ and/or complex spin textures, e.g., skyrmion bubbles^17^, have generated interest in such materials, especially for the role of correlated topological states in the emergent electronic properties. Here we present a large AHE in CoNb_3_S_6_ that cannot be understood in terms of conventional mechanisms of the AHE.

CoNb_3_S_6_ is a member of a large class of intercalated transition metal dichalcogenides, where a 3*d*-transition metal sandwiches between layers of a 5*d*-transition metal dichalcogenide that are coupled by a weak Van der Waals force. CoNb_3_S_6_ represents the 1/3 fractional intercalation of Co atoms between the layers of NbS_2_^18^. It crystallizes in the hexagonal chiral space group *P*6_3_ 22^19,20^. It orders antiferromagnetically below ~26 K and is known to have a rather complex susceptibility for the magnetic field applied parallel to the *c*-axis^21,22^. At 4 K, neutron diffraction measurement has suggested a collinear antiferromagnetic state in which the spins are in the *ab*-plane pointing along a certain crystallographic axis^20^. By itself, however, such a spin structure cannot give rise to the anomalous Hall effect.

The key finding of this work is a large *c*-axis anomalous Hall effect in the antiferromagnetic CoNb_3_S_6_. Although CoNb_3_S_6_ shows a small, intrinsic ferromagnetic component (≈0.0013 *μ*_B_ per Co) along the *c*-axis, this small moment alone cannot explain the observed size of the AHE. Based on its chiral crystal structure and the calculated band structure, we attribute the AHE in CoNb_3_S_6_ either to the formation of a complex magnetic texture or to an influence of the small intrinsic ferromagnetic moment on the underlying electronic band structure.

## Results

### Crystal structure and physical properties of CoNb_3_S_6_

**Fig. 1:**
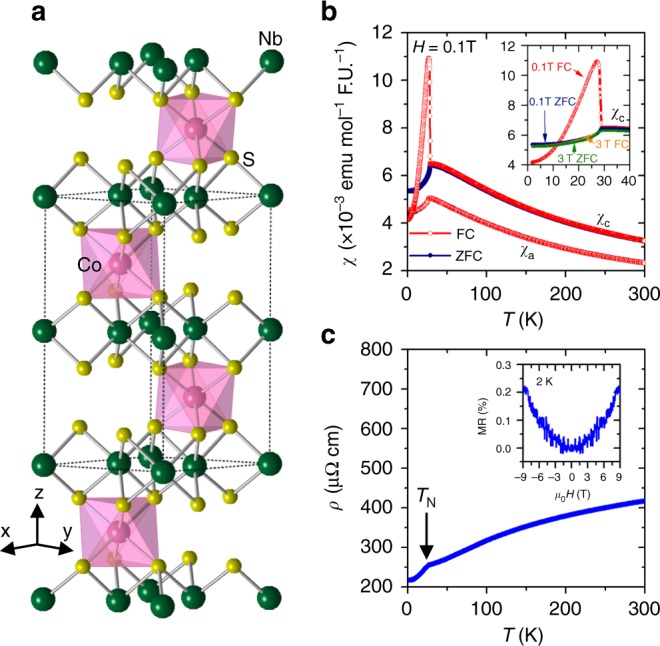
Crystal structure and characteristics of CoNb_3_S_6_. **a** Sketch of crystal structure of CoNb_3_S_6_. Each Co atom is octahedrally coordinated with S atoms. **b** Magnetic susceptibility measured with magnetic field along *a*- and *c*-axis. **c** Electrical resistivity as a function of temperature measured with current along *a*-axis. Inset shows the magnetoresistance defined by MR = (*ρ*_*H*_ − *ρ*_0_)/*ρ*_0_ × 100%, where *ρ*_*H*_ is the resistivity measured at the magnetic field *H* and *ρ*_0_ the resistivity measured at *H* = 0. The MR was measured with current along *a*-axis and magnetic field along *c*-axis

We verified the room temperature crystal structure of CoNb_3_S_6_ in the chiral space group *P*6_3_22 by means of single crystal X-ray diffraction (see Supplementary Note 1 and Supplementary Fig. 1). A sketch of the crystal structure adopted by CoNb_3_S_6_ is shown in Fig. 1a, where the Co atoms occupy the octahedral position between the triangular prismatic layers of the parent compound 2H-NbS_2_. Figure 1b shows the magnetic susceptibility of CoNb_3_S_6_ as a function of temperature measured with a magnetic field of 0.1 T applied along *a*- and *c*-axis (*χ*_*a*_ and *χ*_*c*_, respectively). Over the entire temperature range, *χ*_*c*_ exceeds *χ*_*a*_, consistent with behavior reported in the literature^21^. *χ*_*a*_ shows a kink at *T*_N_ = 27.5 K corresponding to the antiferromagnetic transition and decreases on further cooling down to 1.8 K. The field cooled (FC) and zero field cooled (ZFC) measurements show identical behavior. Along the *c*-axis, the susceptibility below *T*_N_ shows irreversibility between FC and ZFC measurements. The ZFC *χ*_*c*_ shows a behavior similar to that of *χ*_*a*_. However, the FC *χ*_*c*_ increases on cooling below *T*_N_, becomes maximum at 25 K and decreases on further cooling. This increase in FC susceptibility along the *c*-axis near the transition temperature has been reported in previous studies^20,21^ and implies the presence of a small ferromagnetic component along the *c*-axis. With increasing magnetic field, the magnitude of this jump decreases, and at 3 T, FC susceptibility shows a continuous decrease below *T*_N_, as shown in the inset of Fig. 1b, suggesting that the moment along the *c*-axis is suppressed by a sufficiently large magnetic field. At higher temperatures, the susceptibility shows Curie–Weiss behavior. A Curie–Weiss fit to the data between 200 and 300 K (Supplementary Note 2 and Supplementary Fig. 2) yields the powder averaged effective moment of 3.0 *μ*_B_ per Co. This value is smaller than the spin-only moment expected for Co^2+^ (3.87 *μ*_B_) established by neutron diffraction and optical studies^20,23^. Resistivity shows metallic behavior over the measured temperature range of 1.8–300 K, with a sudden drop below 27.5 K (*T*_N_), presumably due to the reduction of electron scattering with the onset of the spin ordering (Fig. 1c). CoNb_3_S_6_ has a very small magnetoresistance of 0.2% at 2 K in the field of 9 T applied along *c*-axis, as shown in the inset of Fig. 1c.

### Magnetization

**Fig. 2:**
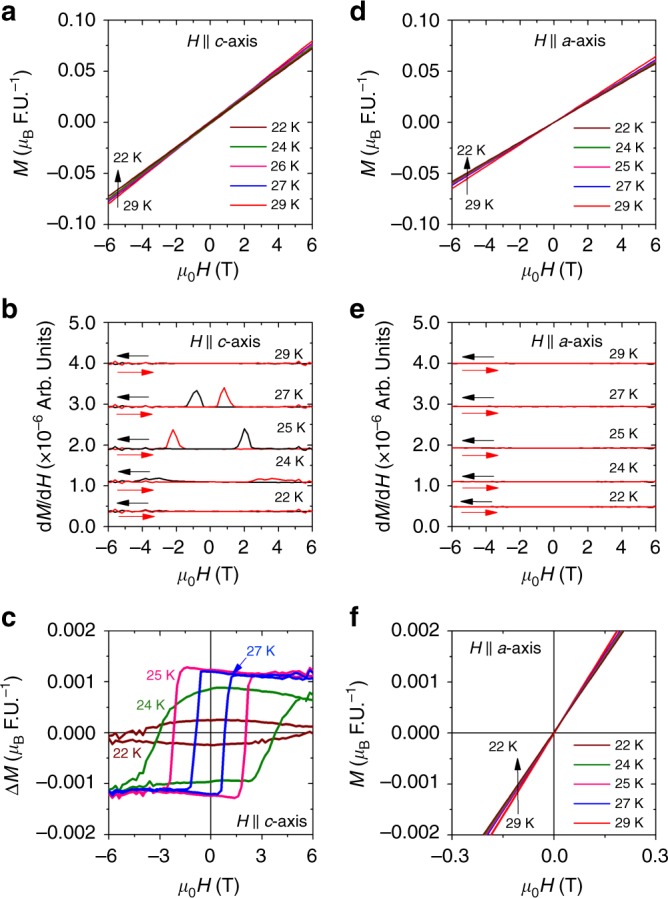
Magnetization of CoNb_3_S_6_. **a** *M* vs *H* measured with magnetic field applied along the *c*-axis. **b** d*M*/d*H* vs *H* for magnetic field applied along the *c*-axis. The black (red) curves represent d*M*/d*H* for decreasing (increasing) *H*. **c** Ferromagnetic moment (Δ*M*) along the *c*-axis obtained by subtracting the antiferromagnetic background from the measured *M* vs *H* presented in **a**. **d** *M* vs *H* for magnetic field applied along the *a*-axis. **e** d*M*/d*H* vs *H* for magnetic field applied along the *a*-axis. The black (red) curves represent d*M*/d*H* for decreasing (increasing) *H*. **f** Low field *M* vs *H* measured with magnetic field along *a*-axis. d*M*/d*H* plots in **b** and **e** are shifted by a constant factor for clarity

*M* vs *H* measured between 29 and 22 K, with the magnetic field along *c*-axis, is shown in Fig. 2a, and the corresponding first-order derivatives are shown in Fig. 2b. Despite the nearly linear *M*–*H* curves, a hysteresis with very small remanent magnetization appears below *T*_N_, which becomes maximum at 25 K (0.0013 *μ*_B_ per formula unit) and decreases at lower temperatures, as shown in Fig. 2b, c. The hysteresis becomes more clear in d*M*/d*H* vs *H* plots shown in Fig. 2b. At 29 K, which is above *T*_N_, d*M*/d*H* is featureless. At 27 K, when decreasing the field from 6 T to −6 T, a peak in d*M*/d*H* appears at −1 T. When increasing the field from −6 T to 6 T, no peak is seen at −1 T. Instead, the peak is observed at 1 T. These peaks appear at the coercive field of the hysteresis in *M* vs *H*. With the decrease in temperature, the coercive field increases and becomes 3 T at 24 K. No such peaks are seen below 24 K. These data suggest that there is an intrinsic, hard ferromagnetic component along the *c*-axis in the ordered state that is flipped by a temperature-dependent critical field. Switching of this ferromagnetic component gives rise to the hysteresis in *M* vs *H*. Below 24 K, a magnetic field of 6 T cannot switch the ferromagnetic component. As a result, no hysteresis is observed. Application of a larger magnetic field is expected to reveal the hysteresis even below 24 K. In fact, this hysteresis is evident in the Hall effect measurement presented below. In Fig. 2c we show the ferromagnetic component as a function of the magnetic field obtained by subtracting a linear antiferromagnetic background from the *M* vs *H* curves presented in Fig. 2a. On the other hand, when magnetic field is applied along the *a*-axis, *M* vs *H* shows linear behavior at all temperatures measured, as depicted in Fig. 2d–f.

### Hall effect

**Fig. 3:**
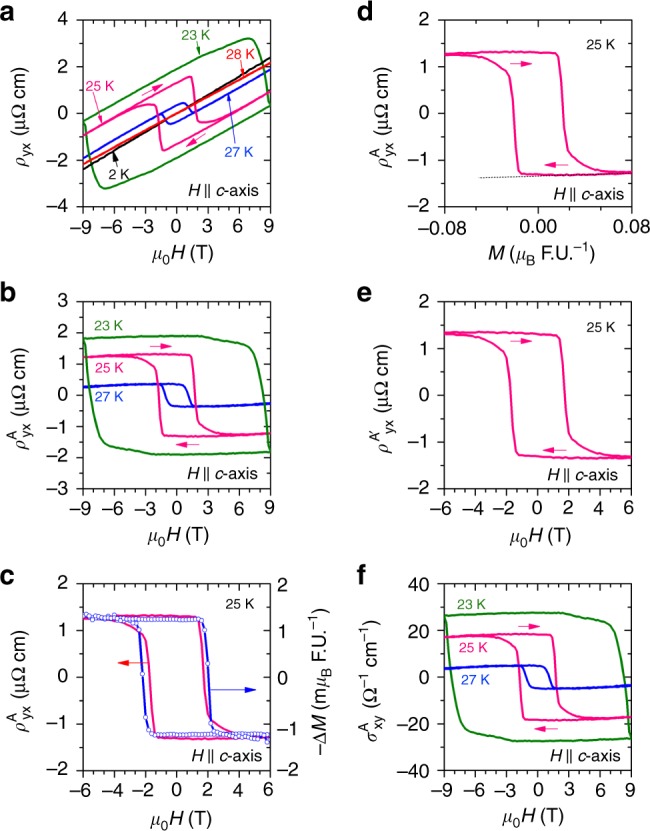
Hall effect in CoNb_3_S_6_ with magnetic field along *c*-axis. **a** Hall resistivity as a function of magnetic field. **b** Anomalous Hall resistivity as a function of magnetic field obtained by subtracting the ordinary Hall resistivity *ρ*_*yx*_ as discussed in the text. **c** Scaling of the Hall resistivity and the ferromagnetic moment. **d** Anomalous Hall resistivity as a function of magnetization. The straight line represents the slope of the high field $\rho _{{\mathrm{yx}}}^{\mathrm{A}}$ vs *M* that gives the anomalous Hall part due to the ferromagnetic component from the field-induced spin tilt. **e** Anomalous Hall resistivity obtained by subtracting the estimated Hall contribution form the ferromagnetic component of magnetic field-induced spin tilt as a function of magnetic field. **f** Hall conductivity as a function of magnetic field

Hall resistivity (*ρ*_*yx*_) vs *H* measured between 28 and 23 K with current along *a*-axis and magnetic field along *c*-axis is depicted in Fig. 3a. The Hall effect at each temperature was measured by cooling the sample in a magnetic field of 9 T and then by changing the magnetic field from 9 to −9 T and subsequently from −9 to 9 T. At 28 K, which is just above *T*_N_, the Hall resistivity is linear, as expected for a normal conductor. The sign of the Hall resistivity indicates that holes are the majority charge carriers in CoNb_3_S_6_. Within the single band model, the estimated carrier concentration (*n* = 1/|*eR*_0_|) is 2.49 × 10^21^ cm^−3^, where *e* is the charge of an electron and *R*_0_ is the ordinary Hall coefficient. When the temperature is lowered below *T*_N_, an additional contribution appears in the Hall resistivity. At 27 K, this anomalous Hall signal is small. It increases both in magnitude and in field with decreasing temperature, becoming maximum at 23 K. Below 23 K, no switching behavior of the Hall resistivity is observed when measured with the maximum field of 9 T. However, the Hall resistivity increases down to 20 K (Supplementary Note 3 and Supplementary Fig. 3). At 15 K, a very small AHE is observed, and at 2 K there is no anomalous Hall effect. These observations reveal two important points. (1) Hysteresis in the AHE is observed when the magnetic field switches the small FM component (Fig. 2a–c). (2) The AHE is observed due to the stabilization of the FM component, which is observed between *T*_N_ and 23 K. At 20 K, a magnetic field of 9 T cannot switch the FM component, and hence the AHE does not switch sign, but gives the same large value. At 2 K, a magnetic field of 9 T cannot induce the FM component at all. As a result, there is no AHE observed for |*H*| ≤ 9 T.

Conventionally, the Hall resistivity in a ferromagnet is given by the relation $\rho _{yx} = R_0B + \rho _{{\mathrm{yx}}}^{\mathrm{A}}$, where *R*_0_ is the ordinary Hall coefficient, $B = \mu _0H,\,{\mathrm{and}}\,\rho _{{\mathrm{yx}}}^{\mathrm{A}}$ is the anomalous Hall resistivity^1^. In Fig. 3a, we see that the Hall resistivity measured in the paramagnetic state, i.e., at 28 K and in the antiferromagnetic state, except near the switching fields has the same slope. This indicates that *R*_0_*B* is constant between 28 and 23 K, and allows us to subtract the ordinary Hall resistivity to obtain the anomalous part. Figure 3b shows $\rho _{{\mathrm{yx}}}^{\mathrm{A}}$ vs *H*, where $\rho _{{\mathrm{yx}}}^{\mathrm{A}}$ is obtained by subtracting the data measured at 28 K from *ρ*_*yx*_ measured below *T*_N_. As there is a finite ferromagnetic component along the *c*-axis, we first examine the effect of this small ferromagnetic component on the anomalous Hall effect. For a ferromagnet, $\rho _{{\mathrm{yx}}}^{\mathrm{A}} = R_{\mathrm{S}}\mu _0M$, where *R*_S_ is the anomalous Hall coefficient, *μ*_0_ is the permeability, and *M* is the magnetization. *R*_S_ scales the *M*–*H* curve to the anomalous part of the Hall resistivity. To check for this scaling in CoNb_3_S_6_, we obtained the magnetization of the ferromagnetic component by subtracting a straight line from the *M* vs *H* curve measured at a representative temperature of 25 K, which shows the maximum remanent magnetization. The obtained magnetization multiplied by −1, (−Δ*M*) vs *H* is plotted in Fig. 3c. The anomalous Hall resistivity measured at 25 K is also plotted in the same figure. These plots show that the anomalous Hall effect does scale with magnetization. *R*_S_ is negative and has an exceedingly large value compared to *R*_0_. *R*_S_ estimated from this scaling at 25 K is −1.41 × 10^−4^ m^3^ C^−1^, which is four orders of magnitude larger than *R*_0_ = 2.4 × 10^−9^ m^3^ C^−1^. In a canonical ferromagnet-like Fe^24^ or ferromagnets with closely related and even the same crystal structure as CoNb_3_S_6_, such as Fe_1/4_ TaS_2_^25^ and Fe_1/3_TaS_2_^26^, *R*_S_/R_0_ ≈ 100, two orders of magnitude smaller than that in CoNb_3_S_6_. This significant boost of the coefficient of AHE in CoNb_3_S_6_ is unlikely to be accounted for by the effect of the weak *c*-axis ferromagnetic component alone and therefore must have an additional contribution.

The contribution of the ferromagnetic component to the anomalous Hall resistivity, i.e., *R*_S_ can be estimated from the high magnetic field part of the anomalous Hall resistivity vs magnetization plot. As $\rho _{{\mathrm{yx}}}^{\mathrm{A}} = R_{\mathrm{S}}\mu _0M$, the slope of $\rho _{{\mathrm{yx}}}^{\mathrm{A}}$ vs *M* gives *μ*_0_*R*_S_. In Fig. 3d, we see that $\rho _{{\mathrm{yx}}}^{\mathrm{A}}$ vs *M* resembles $\rho _{{\mathrm{yx}}}^{\mathrm{A}}$ vs *H* such that $\rho _{{\mathrm{yx}}}^{\mathrm{A}}$ changes sign at the coercive fields. After the abrupt sign change, $\rho _{{\mathrm{yx}}}^{\mathrm{A}}$ shows a linear variation with *M*. This linear behavior of $\rho _{{\mathrm{yx}}}^{\mathrm{A}}$ vs *M* at high field arises from the continuous spin tilting by the magnetic field toward the *c*-axis and represents the contribution from the field-induced ferromagnetic component. The slope in this linear regime gives *μ*_0_*R*_S_ for this compound, which is represented by dashed lines in Fig. 3d. *R*_S_ estimated from this slope is −1.12 × 10^−7^ m^3^ C^−1^, which is comparable to that of the ferromagnet Fe_1/3_TaS_2_^26^. The extra component in the anomalous Hall resistivity, which we label as $\rho _{{\mathrm{yx}}}^{{\mathrm{A}}^\prime }$ can be obtained by subtracting this linear part from the anomalous Hall effect: $\rho _{{\mathrm{yx}}}^{{\mathrm{A}}^\prime } = \rho _{{\mathrm{yx}}}^{\mathrm{A}} - R_{\mathrm{S}}\mu _0M$ and is shown in Fig. 3e, which shows that contribution from the FM component from spin tilting is negligibly small compared to $\rho _{{\mathrm{yx}}}^{\mathrm{A}}$. In Fig. 3f, we plot the anomalous Hall conductivity $\sigma _{{\mathrm{xy}}}^{\mathrm{A}} = \rho _{{\mathrm{yx}}}^{\mathrm{A}}/\left[ {\left( {\rho _{{\mathrm{yx}}}^{\mathrm{A}}} \right)^2 + \left( {\rho _{{\mathrm{xx}}}} \right)^2} \right]$ as a function of magnetic field between 27 and 23 K. The anomalous Hall conductivity at 23 K takes the value 27 Ω^−1^ cm^−1^. A similar behavior, in which a very small ferromagnetic component and its anomalously large scaling with the anomalous Hall resistivity, has been observed in Mn_3_Sn and Mn_3_Ge. Here, the anomalous Hall effect has been attributed to the combined effect of non-collinear antiferromagnetic spin texture and electronic band structure^12,13^. A large anomalous Hall coefficient, reported for Fe_3_Sn_2_^15,16^ cannot be described by the ferromagnetic component alone and is believed to be due to the massive quasi-2D Dirac cones near the Fermi energy. Likewise, the large AHE observed in frustrated systems such as Pr_2_Ir_2_O_7_ has been attributed to the non-coplanar spin texture^27^.

## Discussion

We now discuss possible origins of the anomalous Hall effect in CoNb_3_S_6_. First, we can rule out impurity-related extrinsic mechanisms of the AHE from the fact that the AHE vanishes at low temperatures, where the laboratory magnetic fields are insufficient to alter spins state, e.g., at 2 K as shown in Fig. 3a and Supplementary Fig. 3a. Second, a collinear antiferromagnetic state cannot give rise to the anomalous Hall effect. Finally, we showed above that an FM component due to simple magnetic field-induced spin tilt alone cannot account for the observed AHE. We thus consider other scenarios for the origin of the AHE in CoNb_3_S_6_: (1) non-collinear, non-coplanar or other complex magnetic textures, or (2) interplay of electronic and magnetic degrees of freedom.

CoNb_3_S_6_ has a chiral crystal structure. In a magnet with such a chiral lattice, competition among the exchange interaction, Dzyaloshinskii–Moriya interaction (DMI), magnetocrystalline anisotropy, and Zeeman energy may result in complex magnetic textures^28–30^, including non-collinear or non-coplanar spins that can give rise to large AHE^10–13,27,31^. The neutron diffraction experiment reported by Parkin et al.^20^ revealed no such complex spin structure. However, this is not unexpected given (1) the measurement is carried out in zero field at 4 K, where our data does not show AHE when measured up to the magnetic field of 9 T, and (2) a diffraction experiment such as that carried out by Parkin et al. would be insensitive to the large-scale spin structures. Thus, the nature of the spin structure in CoNb_3_S_6_ near *T*_N_ remains an open question.

**Fig. 4:**
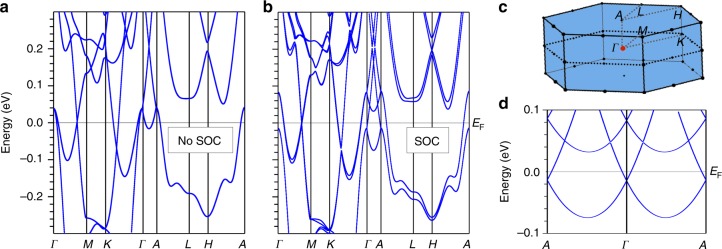
Electronic band structure of CoNb_3_S_6_. **a** Without inclusion of spin–orbit coupling and **b** with inclusion of spin–orbit coupling. Spin–orbit coupling causes splitting of the bands, which is most pronounced along *Γ*−*A*. **c** Brillouin zone of CoNb_3_S_6_. **d** Electronic band structure (with SOC) near the Fermi energy along the *A*–*Γ*–*A* high symmetry line

Figure 4a–c shows the paramagnetic band structure of CoNb_3_S_6_ without inclusion of SOC. The metallic character of CoNb_3_S_6_ is evident by the presence of the hole pockets along *Γ*−*A*, which is consistent with the measured hole-like character of the charge carriers. There are two linear electron and hole band crossings along *Γ*−*M* and *K*−*Γ*. Inclusion of SOC (Fig. 4b) opens a small gap at the band crossings along these lines and splits the bands due to the lack of inversion symmetry^32,33^. The band structure along *Γ*−*A* is enlightening in this regard. In the calculation with SOC (Fig. 4a) both at *Γ* and *A*, two doubly degenerate bands touch at a point ≈40 meV above the Fermi energy (*E*_F_). The splitting of these bands due to SOC along *Γ*−*A* (Fig. 4b) is remarkably large and results in the formation of several linearly crossing bands and avoided crossings. In Fig. 4d, we show the band dispersion along *A*–*Γ*–*A* near *E*_F_ (see Supplementary Note 4 and Supplementary Fig. 4 for symmetry analysis). Bands cross linearly at all of these points. Two of these points lie within ≈15 meV of *E*_F_. It has been established rigorously that in non-magnetic crystals and in directions parallel to a 6_3_ screw axis (along *Γ*−*A* in CoNb_3_S_6_), band degeneracies at the *Γ* points are Weyl nodes^34^. The AHE in CoNb_3_S_6_ is observed only when the external magnetic field induces a small moment along *c*-axis, which suggests that the large enhancement in AHE may result due to the combined effect of such a field in the presence of the near *E*_F_ Weyl nodes.

In summary, CoNb_3_S_6_ is a member of the class of antiferromagnets that show a large anomalous Hall effect. The large AHE is not explained by the reported collinear antiferromagnetic structure, and we suggest the formation of complex, noncollinear magnetic textures or an interplay between the magnetic texture and electronic band structure as two possible mechanisms for the large AHE. CoNb_3_S_6_ is a member of a large family of the 1/3 intercalated TX_2_ compounds, where T is a 4- or 5-*d* transition metal element, and X is a chalcogen (S, Se). As the change in the intercalated 3*d* element changes the nature of both magnetic and electronic structure, our realization of the AHE in CoNb_3_S_6_ opens a platform to explore and perhaps manipulate the interplay among spin texture, electronic band structure, and the associated emergent phenomena in a large class of poorly explored materials.

## Methods

### Crystal growth and characterization

Single crystals of CoNb_3_S_6_ were grown by chemical vapor transport using iodine as the transport agent. First, a polycrystalline sample was prepared by heating stoichiometric amounts of cobalt powder (Alfa Aesar 99.998%), niobium powder (Johnson Matthey Electronics 99.8%), and sulfur pieces (Alfa Aesar 99.9995%) in an evacuated silica ampoule at 900 °C for 5 days. Subsequently, 2 g of the powder was loaded together with 0.5 g of iodine in a fused silica tube of 14 mm inner diameter. The tube was evacuated and sealed under vacuum. The ampoule of 11 cm length was loaded in a horizontal tube furnace in which the temperature of the hot zone was kept at 950 °C and that of the cold zone was ≈850 °C for 7 days. Several CoNb_3_S_6_ crystals formed with a distinct, well-faceted flat plate-like morphology. The crystals of CoNb_3_S_6_ were examined by single crystal X-ray diffraction at beamline 15-ID-D at the APS, Argonne National Laboratory (ANL), where the data were collected with an APEX2 Area Detector using synchrotron radiation (*λ* = 0.41328 Å) at 293 K. Compositional analysis was done using an energy dispersive X-ray spectroscopy (EDS) at the Electron Microscopy Center, ANL.

### Magnetic and transport property measurements

Magnetization measurements were made using a Quantum Design VSM SQUID. Both in FC and ZFC mode, susceptibility data were measured by sweeping temperature up from 1.8 K. In the derivative of *M* vs *H*, we observed a small peak around *H* = 0 both above and below *T*_N_ and both along *a*- and *c*-axis, possibly due to an unknown paramagnetic impurity. For data at all temperatures presented in Fig. 2, a background measured at 30 K was subtracted. A small asymmetry of the peak position in the d*M*/d*H* vs *H* was observed when only one loop of *M* vs *H* was measured. We did not see such an asymmetry when a second loop of the *M* vs *H* was measured, which is presented in Fig. 2. Transport measurements were performed on a quantum design PPMS following a conventional 4-probe method. Au wires of 25 μm diameter were attached to the sample with Epotek H20E silver epoxy. An electric current of 1 mA was used for the transport measurements. In magnetoresistance measurements, the contact misalignment was corrected by field symmetrizing the measured data. The following method was adopted for the contact misalignment correction in Hall effect measurements. The Hall resistance was measured at *H* = 0 by decreasing the field from the positive magnetic field (*R*_*H*+_), where *H* represents the external magnetic field. Again the Hall resistance was measured at *H* = 0 by increasing the field from negative magnetic field (*R*_*H*−_). Average of the absolute value of (*R*_*H*+_) and (*R*_*H*−_) was then subtracted from the measured Hall resistance. The conventional antisymmetrization method was also used for the Hall resistance measured at 28 K (above *T*_N_) and at 2 K (where no anomalous Hall effect was observed), which gave same result as obtained from the former method.

### Electronic structure calculations

The electronic structure calculations were carried out within density functional theory (DFT) using the all-electron, full potential code WIEN2K^35^ based on the augmented plane wave plus local orbital (APW + lo) basis set^36^. The Perdew–Burke–Ernzerhof (PBE) version of the generalized gradient approximation (GGA)^37^ was chosen as the exchange correlation potential. Spin–orbit coupling (SOC) was introduced in a second variational procedure^38^. A dense *k*-mesh of 28 × 28 × 12 was used for the Brillouin zone (BZ) sampling. A *R*_MT_*K*_max_ of 7.0 was chosen for all calculations. Muffin tin radii were 2.5 a.u. for Nb, 2.45 a.u. for Co, and 2.01 a.u. for S.

### Data availability

The authors declare that the main data supporting the findings of this study are available within the article and its Supplementary Information files. Extra data are available from the corresponding author on request.

## Electronic supplementary material

Supplementary Information
