# Supplementary material for: Large anomalous Hall effect in the chiral-lattice antiferromagnet CoNb3S6
Source: Nat Commun. 2018 Aug 16;9:3280. doi: 10.1038/s41467-018-05756-7 (PMC6095917; doi:10.1038/s41467-018-05756-7)
Supplement: Supplementary file 1 — Supplementary Information [file 41467_2018_5756_MOESM1_ESM.pdf]

Supplementary Information

Large anomalous Hall effect in the chiral-lattice antiferromagnet $CoNb_3S_6$

Ghimire et al.

Supplementary Note 1: Crystal structure and chemical composition

The structure of various intercalated NbS_2 complexes has been investigated in great detail[1]. Characteristic of the diffraction pattern of the 1/3 intercalated compounds is the formation of the 1/3-order superlattice peaks that are clearly observed in the single crystal X-ray diffraction pattern shown in Supplementary Fig. 1, confirming the structure of $CoNb_3S_6$ in the hexagonal space group $P6_322$. We found the lattice parameters of $CoNb_3S_6$ to be $a = b = 5.7862(2)$ and $c = 11.9015(4)$ Å. We determined the chemical composition using energy dispersive X-ray spectroscopy (EDS). An atomic percentage ratio of Co:Nb:S = 10.96:31.23:57.81 was obtained, which gives a composition of $Co_{1.05}Nb_3S_{5.55}$, that is within the expected uncertainty of 5 %.

Supplementary Note 2: Curie-Weiss fit and sample dependence of magnetic susceptibility

Polycrystalline inverse susceptibility (χ_{poly}) of $CoNb_3S_6$ obtained after subtracting a temperature independent constant term χ_0 from the susceptibility - $1/(\chi-\chi_0)$ is plotted in Supplementary Fig. 2(a). χ_{poly} was calculated from the susceptibility measured along a- (χ_a) and c- axis (χ_c) as : $\chi_{\mathrm{poly}} = (2/3)\chi_a + (1/3)\chi_c$. χ_0 was then determined by fitting the data between 150 and 300 K to the equation $\chi = \chi_0 + C/(T - \theta_{\mathrm{CW}})$, where, $C = N_{\mathrm{A}}\mu_{\mathrm{eff}}^2/3k_{\mathrm{B}}$ is the Curie constant and θ_{CW} is the Curie-Weiss temperature. The value of χ_0 is 1.82×10^{-4} emu mol^{-1}F.U.$^{-1}$ A Curie-Weiss fit of the form $\chi - \chi_0 = C/(T - \theta_{\mathrm{CW}})$ to the data between 150 and 300 K was then used to determine the effective moment (μ^{eff}) and θ_{CW}. The values obtained are $\theta_{\mathrm{CW}} = -143$ K and $\mu_{\mathrm{eff}} = 3.0\ \mu_{\mathrm{B}}$ per Co. The effective moment is smaller than the spin only moment of Co^{2+} of 3.78 μ_{B} inferred to be

the valence state of Co in $CoNb_3S_6$ by neutron and optical studies[2,3]. Supplementary Figure 2(b) shows the c-axis susceptibility measured on a second sample, which shows that the FC χ_c below the maximum exhibits some sample-to-sample variation [see Fig.1(b) in main text].

Supplementary Note 3: Hall effect measured in zero field-cool mode

Supplementary Figure 3(a) shows the Hall resistivity of $CoNb_3S_6$ measured in another sample. The magnetic field was applied along c-axis, and the current was along a-axis. For the measurement at each temperature, the sample was cooled from 200 K to that particular temperature in the absence of external magnetic field. The measurement was then carried out by increasing the field from 0 to 9 T. Then from 9 T to -9 T and finally from -9 T to 9 T. The arrows indicate the direction of increasing or decreasing magnetic field. At 30 K, which is above T_N of 27.5 K, and at 2 K, all the measurements fall on the same straight line. At 23 K, a clear hysteresis with coercive field of $\approx$ 8 T is observed. Such a hysteresis with smaller coercive fields is also observed between 27 and 23 K [not shown, see Fig. 3(a) in the main text]. At 20 K, a full hysteresis is not observed, but it still shows an AHE larger than that at 23 K. At 15 K, the behavior is similar to that at 20 K, but with a negligibly small AHE. No AHE is observed at 2 K. Supplementary Figure 3(b) shows the anomalous Hall resistivity obtained by subtracting the normal Hall component (Hall resistivity measured at 30 K) from the Hall resistivity measured at the individual temperature. In this case, the field-cooled Hall resistivity at zero magnetic field at each temperature is subtracted to account for the offset voltage.

Supplementary Note 4: Symmetry analysis

Supplementary Figure 4 shows the calculated DFT band structure with inclusion of spin-orbit coupling along the A-$\mathit{\Gamma}$-A high symmetry line. Symmetry analysis of the bands shows that at A, two doubly degenerate representations, A_9 and A_8 are found close to E_F, while at $\mathit{\Gamma}$ two doubly degenerate $\mathit{\Gamma}_9$ representations are found. The degeneracy at A arises from bands 'sticking' together as a consequence of the 6_3 nonsymmorphic symmetry element. As discussed in the discussion section of the main text, those at $\mathit{\Gamma}$ are Weyl nodes. Compatibility relationships along the $\mathit{\Gamma}$-A line (right panel of Supplementary Fig. 4) show all singly-degenerate bands of $\mathit{\Delta}_{10}$, $\mathit{\Delta}_{11}$, and $\mathit{\Delta}_{12}$ symmetry. An avoided crossing of two $\mathit{\Delta}_{11}$ bands is observed close to $\mathit{\Gamma}$. In the neighborhood of E_F, one accidental degeneracy ($\mathit{\Delta}_{11}$ and $\mathit{\Delta}_{12}$ bands crossing) and four symmetry protected degeneracies are found. Bands cross linearly at all of these points. Two of these points, A_9 and $\mathit{\Gamma}_9$ lie within $\approx$15 meV of E_F.

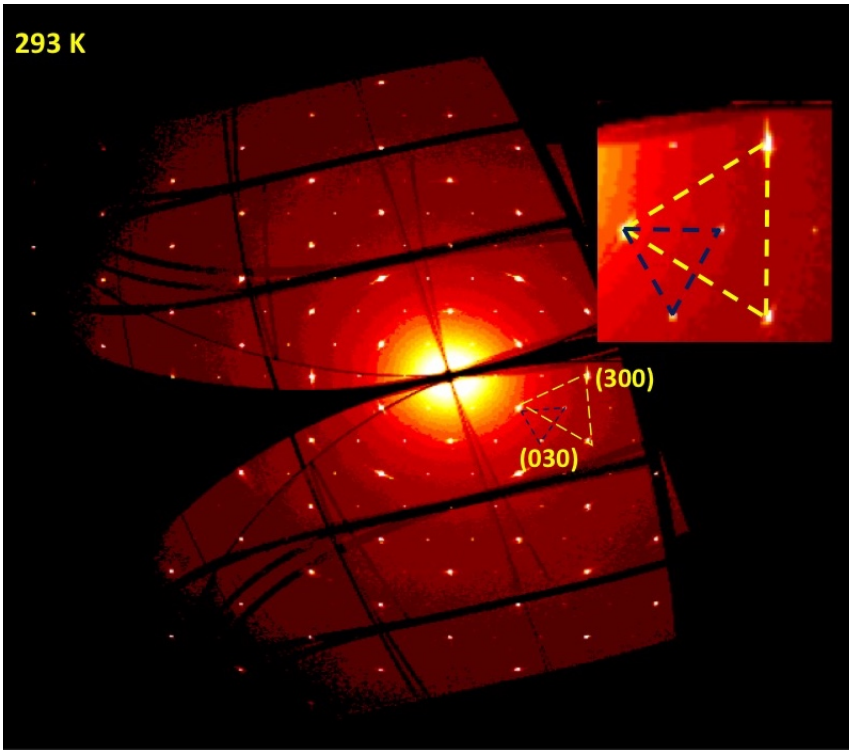

Supplementary Figure 1: X-ray diffraction on a single crystal of $CoNb_3S_6$. Precession image in the *hk0* plane of $CoNb_3S_6$ obtained by single crystal X-ray diffraction collected at 293 K shows the 1/3-order superlattice peaks. The basic hexagonal structure of $CoNb_3S_6$ and the superstructure of NbS_2 are indicated by blue and yellow dotted triangles, respectively. An enlarged view showing the triangles is presented in the inset. The lattice parameters obtained from the refinement of the X-ray pattern at 293 K are: $a = b = 5.7862(2)$ and $c = 11.9015(4)$ Å.

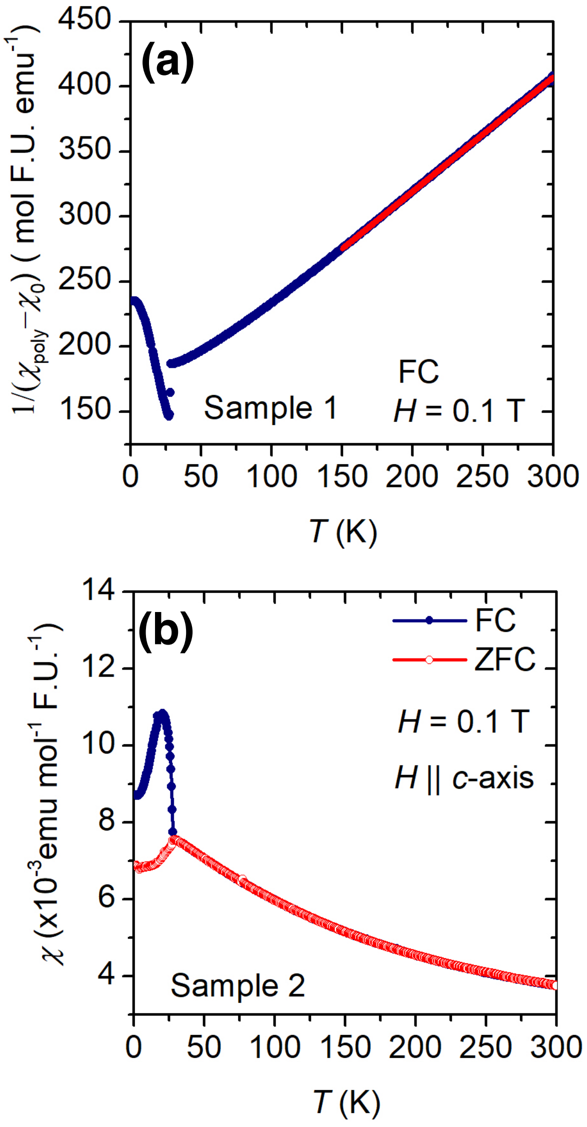

Supplementary Figure 2: DC magnetic susceptibility of $CoNb_3S_6$. (a) Polycrystalline inverse susceptibility obtained after subtracting a temperature independent term χ_0 as a function of temperature. The solid red line is the Curie-Weiss fit to the data. (b) Susceptibility of a second sample of $CoNb_3S_6$ measured with magnetic field applied along the *c*-axis.

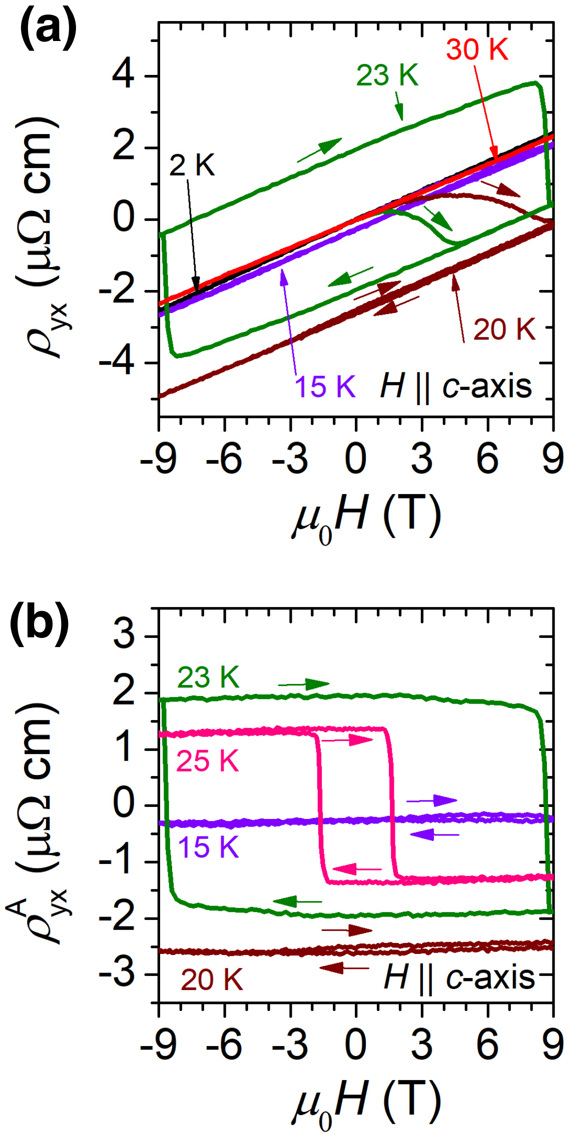

Supplementary Figure 3: Hall effect $CoNb_3S_6$ with magnetic field along the *c*-axis measured first by cooling in absence of external magnetic field. a) Hall resistivity as a function of magnetic field. b) Anomalous Hall resistivity as a function of magnetic field obtained by subtracting the ordinary Hall resistivity ρ_{yx} as discussed in Supplementary Note 3.

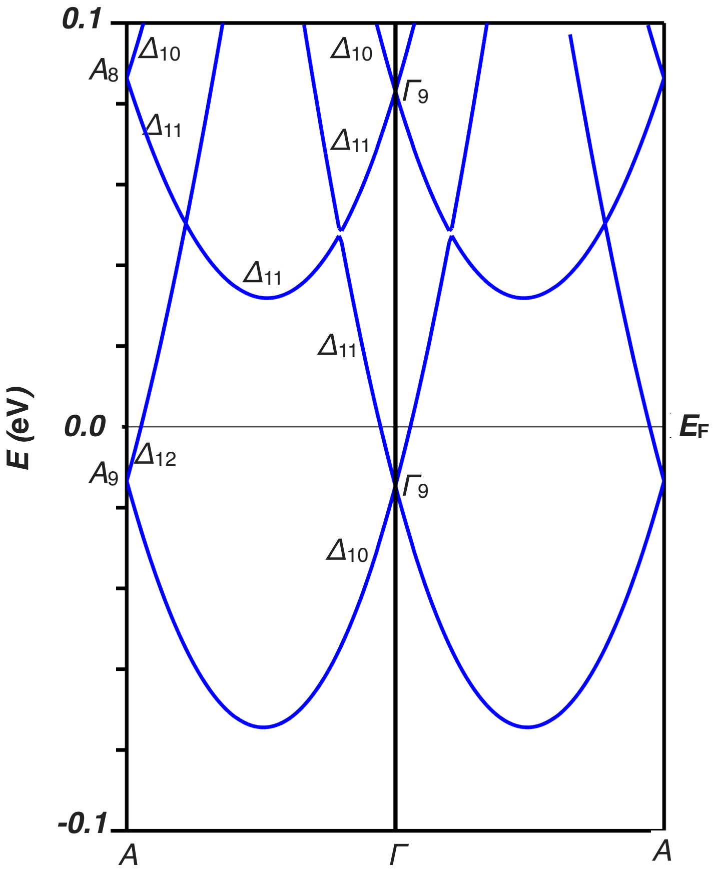

$\Gamma_1(1) \rightarrow \Delta_1(1)$
$\Gamma_2(1) \rightarrow \Delta_1(1)$
$\Gamma_3(1) \rightarrow \Delta_2(1)$
$\Gamma_5(2) \rightarrow \Delta_3(1) \oplus \Delta_5(1)$
$\Gamma_6(2) \rightarrow \Delta_4(1) \oplus \Delta_6(1)$
$\Gamma_7(2) \rightarrow \Delta_7(1) \oplus \Delta_8(1)$
$\Gamma_8(2) \rightarrow \Delta_9(1) \oplus \Delta_{12}(1)$
$\Gamma_9(2) \rightarrow \Delta_{10}(1) \oplus \Delta_{11}(1)$

$A_1(2) \rightarrow \Delta_1(1) \oplus \Delta_2(1)$
$A_2(2) \rightarrow \Delta_3(1) \oplus \Delta_6(1)$
$A_3(2) \rightarrow \Delta_4(1) \oplus \Delta_5(1)$
$A_4(1) \rightarrow \Delta_7(1)$
$A_5(1) \rightarrow \Delta_7(1)$
$A_6(1) \rightarrow \Delta_8(1)$
$A_7(1) \rightarrow \Delta_8(1)$
$A_8(2) \rightarrow \Delta_9(1) \oplus \Delta_{11}(1)$
$A_9(2) \rightarrow \Delta_{10}(1) \oplus \Delta_{12}(1)$

Supplementary Figure 4: Symmetry analysis of electronic bands of $CoNb_3S_6$. Electronic band structure of $CoNb_3S_6$ calculated with SOC near the Fermi energy along the A-Γ-A high symmetry line. Compatibility relationships joining irreducible representations are listed on the right.

Supplementary References

1. Beal, A. R. in: *Intercalated Layered Materials* Vol. 6 (ed. Lévy, F.) 251-305 (D. Reidel Publishing Company, Dordrecht, 1979).

2. Parkin, S. S. P., Marseglia, E. A. & Brown, P. J. Magnetic structure of $Co_{1/3}NbS_2$ and $Co_{1/3}TaS_2$. *J. Phys. C: Solid State Phys.* **16**, 2765–2778 (1983).

3. Beal, A. R. & Liang, W. Y. Reflectivity spectra of some first row transition metal intercalates of NbS_2. *Philos. Mag.* **33**, 121–131 (1976).
